# ZNF281 Facilitates the Invasion of Cervical Cancer Cell Both In Vivo and In Vitro †

**DOI:** 10.3390/cancers16213717

**Published:** 2024-11-04

**Authors:** Ye Chong, Kun Zhang, Yuting Zeng, Qian Chen, Qian Feng, Nan Cui, Pengsheng Zheng, Litao Ruan, Wei Hua

**Affiliations:** 1 Department of Ultrasound, The First Affiliated Hospital of Xi’an Jiaotong University, Xi’an 710061, China; chong_ye1992@163.com; 2 Department of Pharmacology, School of Pharmacy, Fourth Military Medical University, Xi’an 710032, China; kunzhang1900@163.com; 3 Department of Reproductive Medicine, The First Affiliated Hospital of the Medical College, Xi’an Jiaotong University, Xi’an 710061, China; 13659211769@163.com (Y.Z.); chenqian1006@126.com (Q.C.); fengqian@stu.xjtu.edu.cn (Q.F.); cuin2003@xjtufh.edu.cn (N.C.); zpsheng@mail.xjtu.edu.cn (P.Z.); 4 Department of Obstetrics and Gynecology, Xijing Hospital, Fourth Military Medical University, Xi’an 710032, China

**Keywords:** zinc finger transcription factor ZNF281/ZBP-99 protein, cervical cancer, tumor metastasis, epithelial–mesenchymal transition

## Abstract

**Background**: Cervical cancer is the fourth most common cancer among women worldwide. The zinc finger transcription factor 281 (ZNF281)/ZBP-99 protein specifically binds to GC-rich DNA sequences and regulates gene expression, and it has been shown to promote tumor progression. In this study, we aim to investigate the function and molecular mechanism of ZNF281 in uterine cervical carcinoma. **Methods**: We conducted immunohistochemistry and Western blot assays to determine the expression of ZNF281 in eight human cervical cancer tissues. And, xenograft experiments involving the injection of HeLa cells into nude mice was used to determine the function of ZNF281 on proliferation. Transwell assays were used to detect the migration and invasion of HeLa cells after indicated that ZNF281 overexpression. **Results:** Our results indicated that ZNF281 protein levels were higher in cervical cancer tissues compared to normal cervical tissues. Additionally, ZNF281 was expressed in human cervical carcinoma cell lines, including HeLa, SiHa, C-33 A, CaSki, and HT-3, and is localized in both the cell nucleus and cytoplasm. ZNF281 overexpression did not influence HeLa cell proliferation or tumor size in situ. Moreover, nude mice injected with ZNF281-overexpressing cell lines developed more tumor lesions in the lungs compared to those injected with control cell lines. **Conclusions**: These findings suggest that ZNF281 is associated with tumor metastasis without affecting cell proliferation, both in vivo and in vitro.

## Simple Summary

Although the zinc finger transcription factor 281 (ZNF281)/ZBP-99 protein has been shown to promote tumor progression, its role in the development of cervical cancer remains unclear. We find that its level is higher in cervical cancer tissues than in normal cervical tissues. And it promotes metastasis in HeLa cell lines by facilitating the epithelial-mesenchymal transition process, thereby enhancing the migration of HeLa cells in vivo. Our research might provide a new marker for predicting the development of cervical cancer.

## 1. Introduction

Cervical cancer (CC) is the fourth most common cancer among women worldwide [1]. In China, more than 135,000 women are diagnosed with CC each year, accounting for one-third of the global cases [2]. Low-grade squamous intraepithelial lesion (LSIL) and high-grade squamous intraepithelial lesion (HSIL) are groups of precancerous lesions of uterine cervical epithelium closely related to the development of cervical invasive carcinoma. Numerous studies have identified several factors such as HPV infection, poor hygiene, cervical laceration, and high-risk male partners, adding the risk of LSIL, HSIL, and CC [3]. Epidemiological studies have shown that 99.8% of CC specimens are infected with high-risk human papillomavirus (HPV) subtypes [4]. In addition, studies have found that the infection rate is less than 4% in women without CC but significantly higher in women with LSIL and HSIL [5]. Based on these findings, efforts are being made worldwide to promote cervical exfoliation cytology examination and CC vaccine. As a result of these efforts, a large number of patients with LSIL and HSIL and early cancer have received treatment on time, leading to a significant reduction in the incidence of invasive cancer [6].

The zinc finger transcription factor 281 (ZNF281)/ZBP-99 protein was discovered in a yeast one-hybrid assay and was named GC-box-binding zinc-finger protein (GZP1) because it binds to GC-rich DNA sequences [7,8]. ZBP-99 protein is involved in cell proliferation [9], apoptosis [10], differentiation [11], and tumorigenesis [12]. High levels of ZNF281 have previously been detected in the placenta, kidney, brain, heart, liver, and lymphocytes [8]. The Sp/Kruppel-like family of transcription factors (Sp/KLF), including Sp1 and Sp3, also bind to GC-rich DNA sequences [13,14]. Thus, Sp/KLF and ZNF281 compete for specific DNA-binding sites, forming a complex gene regulatory network [15]. ZNF281 regulates cell stemness in mice by inhibiting Nanog expression through recruiting the NuRD inhibitory complex at the Nanog promoter [16,17]. Knocking out *ZNF281* in mice is fatal to the embryo, indicating its crucial role in development [18]; however, it may also have other important functions in adult cells. Indeed, ZNF281 induces epithelial–mesenchymal transition (EMT) in colon cancer cells by regulating the expression of SNAIL1 and other key genes implicated in EMT [19]. In addition, ZNF281 knockdown promotes osteogenesis of multipotent stem cells [20].

In order to determine the role of ZNF281 protein in the CC tissues and uncover new markers for predicting the development of CC, we detect the effect of ZNF281 on metastasis and proliferation. These results suggest that ZNF281 promotes tumor metastasis without affecting HeLa cell proliferation in vitro or tumor size in animal models, indicating different roles of ZNF281 in metastasis and proliferation of CC cells.

## 2. Materials and Methods

### 2.1. Cell Lines and Culture Conditions

Human normal cervical cell lines (ECT1/E6E7) and human CC cell lines HeLa, SiHa, C-33 A, CaSki, and HT-3 cell lines were purchased from the American Type Culture Collection (ATCC; Manassas, VA, USA) and authenticated by STR profiling. The cell lines were maintained in recommended media supplemented with 10% fetal bovine serum at 37 °C and 5% CO_2_. Dulbecco’s modified Eagle’s medium (DMEM; Sigma-Aldrich, Darmstadt, Germany) was used for HeLa, SiHa, and C-33 A cells; McCoy’s 5A medium (Sigma-Aldrich, Germany) was used for HT-3 cells; and RPMI 1640 (Sigma-Aldrich, Germany) was used for CaSki cells under identical conditions.

### 2.2. Human Tissue Specimens, Immunohistochemistry, and Immunocytochemistry

A total of 16 tissue samples, including 8 NC and 8 squamous cervical cancer (SCC), without a history of chemotherapy, immunotherapy, or radiotherapy, were obtained from the First Affiliated Hospital of Xi’an Jiaotong University Medical College (Xi’an, China) between January 2008 and December 2016, as previously reported [21]. The procedures were approved by the Ethics Committee of the School of Medicine of Xi’an Jiaotong University, China (No. 2014-113). Hematoxylin and eosin (H&E)-stained sections were used for pathological diagnosis, and the International Federation of Gynecology and Obstetrics (FIGO) classification system was used to determine clinical stages. Immunohistochemical (IHC) staining was performed as previously described [21]. Images were captured using Olympus CX31 microscope digital camera and Leica DFC500 digital camera and processed with LAS AF 2.2.0 software (Leica, Solms, Germany). Immunoreactivity scores (IRSs) were determined by two investigators. Staining intensity was scored on a scale from 0 to 3: negative (0), weak (1), moderate (2), or strong (3). The percentage of positive cells, defined as the relative positive-staining area, was scored on a scale from 0 to 4: 0–5% (0), 6–25% (1), 26–50% (2), 51–75% (3), and 76–100% (4). The IRS was calculated by multiplying the “staining intensity score” by the “percentage of positive cells”. IRSs ≤ 2 were defined as ZNF281-negative, whereas IRSs between 3 and 12 (3 ≤ IRS ≤ 12) were defined as ZNF281-positive.

### 2.3. Western Blot

Western blot was performed as previously described [22]. Horseradish peroxidase-conjugated anti-rabbit and anti-mouse IgG were purchased from Thermo Fisher Scientific (New York, NY, USA). The antibodies used were anti-ZNF281 (1:500 dilution, sc-166933; Santa Cruz Biotechnology, Inc., Dallas, TX, USA), anti-E-cadherin (1:500 dilution, sc-8426; Santa Cruz Biotechnology, Inc., Dallas, TX, USA), anti-C-myc (1:500 dilution, sc-40; Santa Cruz Biotechnology, Inc., Dallas, TX, USA), anti-GAPDH (1:500 dilution, sc-47724; Santa Cruz Biotechnology, Inc., Dallas, TX, USA), and anti-vimentin (1:1000 dilution, sc-6260; Santa Cruz Biotechnology, Inc., Dallas, TX, USA). GAPDH was used as the control.

### 2.4. Plasmid Construction and Stable Transfectants

Full-length human ZNF281 (NM_012482.5) cDNA was amplified by reverse-transcription polymerase chain reaction (PCR) using mRNA extracted from HeLa cells. Primer sequences were as follows:

- ZNF281-F: 5′-GGAAGATCTGCCACCATGAAAATCGGCAGTGGGTTCCT-3′;
- ZNF281-R: 5′-ACGCGTCGACTTACCTGTAACTCTGGCTGGTGGGT-3′.

The full-length human ZNF281 coding sequence was cloned into the pIRES2-AcGFP vector (Clontech, Mountain View, CA, USA) via the *Nhe*I and *Sac*I sites. The plasmid was transiently transfected into HeLa cells using Lipofectamine 2000 reagent (Invitrogen, Carlsbad, CA, USA) as reported previously [23]. Stable clones were selected using the G418 reagent (Calbiochem, Darmstadt, Germany) and identified by Western blot. Cell growth curves and MTT assays were performed to assess cell proliferation, whereas migration and invasion assays were performed to evaluate cell motility.

### 2.5. In Vivo Metastasis Experiments

Female BALB/c nude mice (4–6 weeks old) were obtained from Shanghai SLAC Laboratory Animal Co., Ltd. (Shanghai, China) and housed in a specific pathogen-free room with constant temperature (22–25 °C) and humidity (40–50%). For the spontaneous metastasis assay, 12 mice were randomly divided into 2 groups (6 mice each). ZNF282-overexpressed HeLa (HeLa-ZNF281) cells and the respective control (HeLa-GFP) cells (1 × 10^6^/mouse) in the exponential growth phase were injected (100 μL/site) subcutaneously on the dorsum of each mouse. Tumor volume was measured every 2 to 3 days using manual calipers and calculated using the formula: V = 0.5 × length × width^2^. After 8 weeks, the mice were sacrificed, and the lungs were removed for histologic examination [23]. For the experimental lung metastasis assay, 12 mice were randomly divided into 2 groups (6 mice each). After being anesthetized with isoflurane, the mice were injected with HeLa-ZNF281 or HeLa-GFP cells (5 × 10^5^/mouse) through their tail veins. After 6 weeks, the mice were sacrificed and the lungs were removed for histologic examination. The lung tissues were then fixed in 4% formalin and embedded in paraffin and cut into 5 μm sections and stained with H&E. Micrometastases were counted under a light microscope at ×10 magnification. An average of three sections per mouse sample, counted at 50 μm intervals, were considered. All animal studies were approved by the Xi’an Jiaotong University Ethical Committee and conducted in compliance with the guidelines of Laboratory Animal Care Committee (2016-117).

### 2.6. Migration and Invasion Assays

Transwell chambers (pore size: 8 µm; Corning, Corning, NY, USA) were used for cell migration and invasion assays. For migration assay, 8 × 10^4^ HeLa-ZNF281 cells or HeLa-GFP cells in serum-free medium were plated on uncoated inserts and incubated for 48 h. For invasion assay, inserts were coated with 70 μL of 1:8-diluted Matrigel (BD Biosciences, Milpitas, CA, USA), and 1 × 10^5^ cells in serum-free medium were plated and incubated for 48 h. Six hundred microliters of culture medium containing 20% fetal bovine serum (FBS; Invitrogen) were added to the lower chamber. Noninvaded cells were removed, and the cells attached to the bottom of the membrane were fixed with 4% paraformaldehyde, stained with 5% crystal violet (Sigma-Aldrich), and counted at ×200 magnification. The experiments were repeated three times [24].

### 2.7. RNA Isolation and Real-Time PCR

Total RNA was isolated using TRIzol reagent (Invitrogen), and first-strand cDNA was synthesized using the PrimeScript RT Reagent Kit (TaKaRa, Osaka, Japan). cDNA and SYBR Green fluorescence signal-detection assays (TaKaRa) were used for real-time PCR. Data were normalized to the housekeeping gene *GAPDH*. Real-time PCR and data collection were performed on an IQ5 instrument (Bio-Rad, Hercules, CA, USA).

The following primers were used for mRNA analysis:

- *Snail1* forward: 5′-GAACCTGCTGGTGCTATGTGT-3′;
- *Snail1* reverse: 5′-TCGCTGTAGTTAGGCTTCCGATT-3′;
- *Snail2* forward: 5′-ATGCATATTCGGACCCACACATTAC-3′;
- *Snail2* reverse: 5′-AGATTTGACCTGTCTGCAAATGCTC-3′;
- *Zeb1* forward: 5′-TACAGAACCCAACTTGAACGTCACA-3′;
- *Zeb1* reverse: 5′-GATTACACCCAGACTGCGTCACA-3′;
- *Zeb2* forward: 5′-AAATGCACAGAGTGTGGCAAGG-3′;
- *Zeb2* reverse: 5′-CTGCTGATGTGCGAACTGTAGGA-3′;
- *C-myc* forward: 5′-CCTGGTGCTCCATGAGGAGA-3′;
- *C-myc* reverse: 5′-TCCAGCAGAAGGTGATCCAGAC-3′;
- *Gapdh* forward: 5′-GCACCGTCAAGGCTGAGAAC-3′;
- *Gapdh* reverse: 5′-TGGTGAAGACGCCAGTGGA-3′.

Real-time PCRs were quantified using the 2^−ΔΔCT^ method and repeated at least three times.

### 2.8. Cell Growth and Viability Assays

Cells (5 × 10^4^) were seeded with 2 mL of media into 35 mm tissue culture plates for 7 days. Cells were counted every 2 days using a hemocytometer under a light microscope. Cell viability assays were performed by applying 3-(4,5-dimethylthiazole-yl)-2,5-diphenyl tetrazolium bromide (Sigma-Aldrich) dye to cells seeded in 96-well plates at a density of 1000 cells per well for 7 days or 3000 cells per well for 48 h, as described in a standard protocol. Absorbance was measured at 490 nm (Bio-Rad).

### 2.9. Statistical Analyses

Each sample was assayed in triplicate, and each experiment was repeated three times. Data are presented as mean ± SD. Student’s *t*-test (unpaired, 2-tailed) or one-way ANOVA was used to compare the means of independent samples. For repeated measurement data, univariate ANOVA after lower-bound correction was used. Statistical analyses were performed using SPSS for Windows v. 16.0 (SPSS Inc., Chicago, IL, USA). *p* < 0.05 were considered statistically significant.

## 3. Results

### 3.1. ZNF281 Expression in Cervical Cancer Tissues and Cell Lines

**Figure 1:**
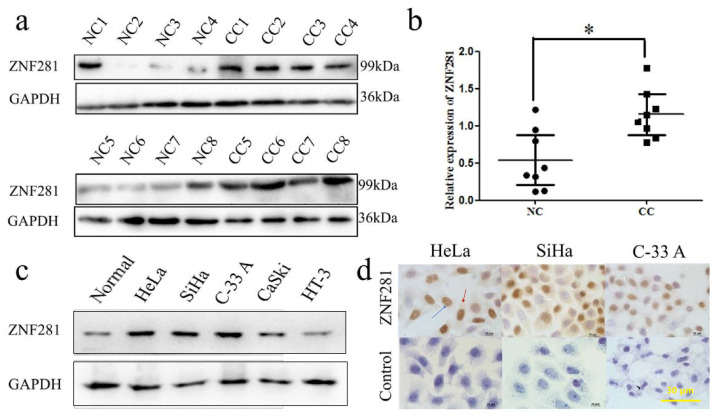
ZNF281 expression is elevated in CC. (**a**) Western blot was used to detect the expression of ZNF281 in normal cervical tissues and CC tissues, and GAPDH was set as the internal reference protein. (**b**) The expression of ZNF281 in NC and CC patients was quantitatively analyzed. (**c**) Western blot analysis of the expression of ZNF281 in human normal cervical cell lines (ECT1/E6E7) and human CC cell lines HeLa, SiHa, C-33A, CaSki and HT-3. (**d**) The expression of ZNF281 in CC cell lines HeLa, SiHa and C-33A was detected by cellular immunocytochemical method. Extensive expression of ZNF281 were found in these three cell lines. The blue arrow and red arrow showed the ZNF281 expression in the nucleus and cytoplasm of HeLa cells, respectively. Statistical analysis was performed using two-tailed unpaired Student’s *t*-test. * *p* < 0.05 versus NC group. Original western blots are presented in Figures S1 and S2.

To determine ZNF281 expression in CC tissues, we randomly selected 8 NC tissues and 8 CC tissues for Western blot analysis. As preliminary data, the expression of ZNF281 was detected by Western Blot (Figure 1a), and was higher in CC tissues than in negative control (NC) tissues (Figure 1b). Similarly, we also found that ZNF281 had higher expressions in HeLa, SiHa, and C-33 A CC cell lines compared with CaSki and HT-3 (Figure 1c). IHC showed that in HeLa, SiHa, and C-33A cells, ZNF281 was mainly located in the cytoplasm and nucleus (Figure 1d). These findings suggest that ZNF281 may play a role in the occurrence and development of CC.

### 3.2. Construction of ZNF281-Overexpressing HeLa Cell

**Figure 2:**
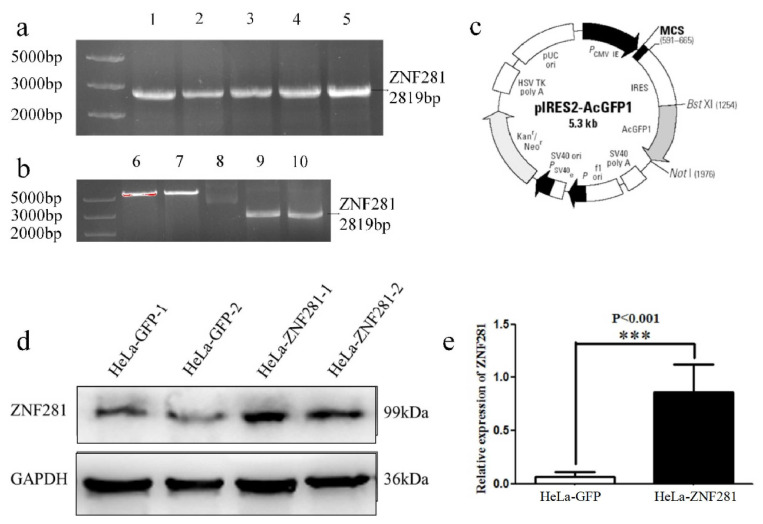
Construction of HeLa cell line overexpressing ZNF281. (**a**) PCR amplification of coding sequence (CDS) region of ZNF281 gene. Lanes 1–5 were the target segments after amplification. (**b**) Construction and identification of eukaryotic expression plasmid pIRES2-AcGFP-ZNF281. Lanes 6 and 7 were plasmid pIRES2-AcGFP1-Neo after double digestion. Lane 8 was plasmid pIRES2-AcGFP1-Neo; Lane 9 and 10 were the target segments after the double digestion reaction. (**c**) Structure diagram of plasmid pIRES2-AcGFP1-Neo. (**d**) Western blot identification of positive clones. (**e**) Quantitative analysis of ZNF281 expression in HeLa-ZNF281 cells and HeLa-GFP cells. Statistical analysis was performed using two-tailed unpaired Student’s *t*-test. *** *p* < 0.001. Original western blots are presented in Figure S3.

We chose HeLa cell line as the experimental model to study the role of ZNF281 in CC cells. Plasmids pIRES2-AcGFP1-Neo (empty) or pIRES2-AcGFP1-ZNF281 (overexpressing) were designed (Figure 2a–c) and transfected into HeLa cells using the cationic liposome method. Successfully transfected cells showed green fluorescence under a fluorescence microscope and were screened using G418 to obtain positive cells with stable ZNF281 expression. Overexpression of ZNF281 protein in these clones was confirmed using Western blot (Figure 2d,e).

### 3.3. Effect of ZNF281 Overexpression in HeLa Cells Proliferation or Tumor Size

**Figure 3:**
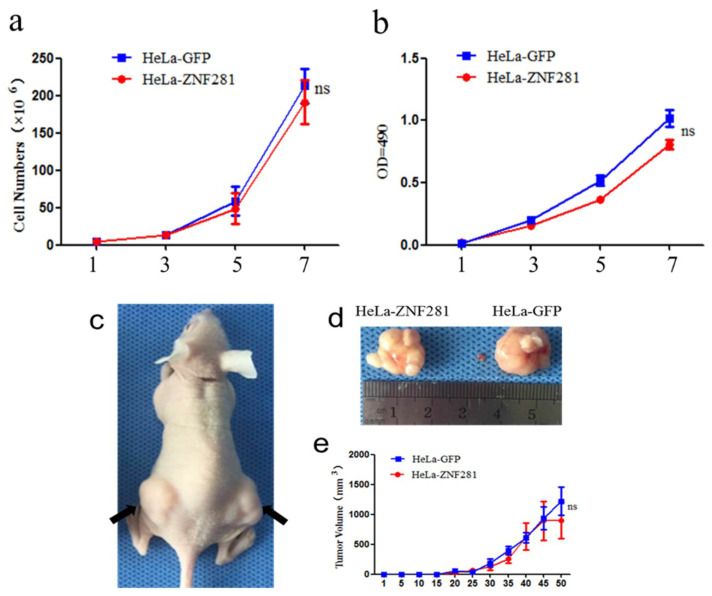
ZNF281 overexpression did not affect HeLa cells proliferation and transplanted tumor size in nude mice. (**a**) Cell counting experiments to detect HeLa-GFP cells and HeLa-ZNF281 cells proliferations under microscope. (**b**) MTT assay to detect HeLa-GFP cells and HeLa-ZNF281 cells proliferations. (**c**) Hela-ZNF281 and Hela-GFP were injected subcutaneously into nude mice. Hela- ZNF281 cells were injected on the left dorsal side of nude mice, and HELA-GFP cells were injected on the right side (black arrows indicated the injected sites). (**d**) At the end of the experiment, subcutaneous transplanted tumors were extracted from the nude mice. (**e**) Growth curve of transplanted tumor. Statistical analysis was performed using univariate ANOVA after lower-bound correction as repeated-measurement data. ns, not significant.

We carried out cell counting and MTT assay to detect the proliferation rate of HeLa-GFP and HeLa-ZNF281 cells, respectively. We observed no difference in cell numbers (Figure 3a) or optical densities between HeLa-GFP and HeLa-ZNF281 cells at 1, 3, 5, and 7 days (Figure 3b). These results indicate that ZNF281 does not affect the proliferation of CC cells in vitro. In addition, we evaluated the effect of ZNF281 on tumorigenesis in vivo by subcutaneously inoculating HeLa-ZNF281 and HeLa-GFP cells into two different hind limbs of the same nude mouse (Figure 3c). Tumor sizes were measured every 4 days for 50 days (Figure 3d). At the end of the experiment, mice were sacrificed, and the sizes of transplanted tumors were measured (Figure 3e). No significant difference in tumor growth rate was found between HeLa-ZNF281 and HeLa-GFP cells, suggesting that ZNF281 did not influence tumor growth in vivo.

### 3.4. Effect of ZNF281 Overexpression in HeLa Cell Migration and Invasion

**Figure 4:**
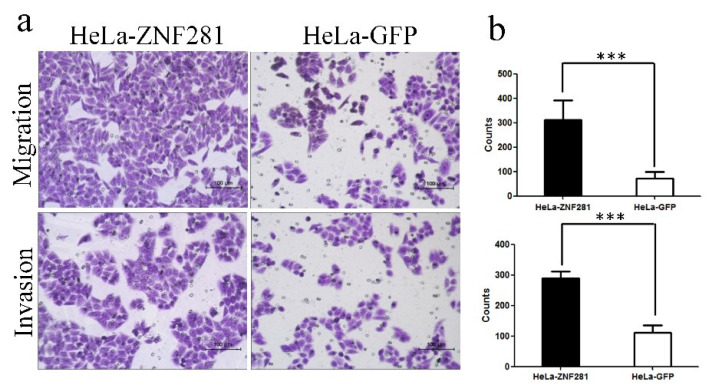
ZNF281 promoted migration and invasion of cervical cancer cells in vitro. (**a**) Transwell migration and invasion test of HeLa-ZNF281 and HeLa-GFP cells in vitro, crystal violet staining results. (**b**) In transwell migration and invasion experiment, the number of cells exiting the cell compartment of HeLa-ZNF281 and HeLa-GFP cells. Statistical analysis was performed using two-tailed unpaired Student’s *t*-test. *** *p* < 0.001.

Transwell assays were used to observe the effect of ZNF281 in the migration and invasion of cancer cells. The basement membrane of the cell was hydrated with a serum-free medium in the migration experiment or hydrated with matrix gel in the invasion experiment. HeLa-GFP and HeLa-ZNF281 cells at the logarithmic growth stage were inoculated into Transwell with 6 × 10^4^ cells per well using a serum-free medium to adjust their cell concentrations. After 48 h, cells were stained using crystal violet and counted. Using the Transwell assay, we found that HeLa-ZNF281 cells showed significantly higher migration and invasion rates compared with HeLa-GFP cells (Figure 4a,b), indicating that ZNF281 promotes the migration and invasion of CC cells in vitro.

### 3.5. Effect of ZNF281 Overexpression on Lung Metastasis in Nude Mice

**Figure 5:**
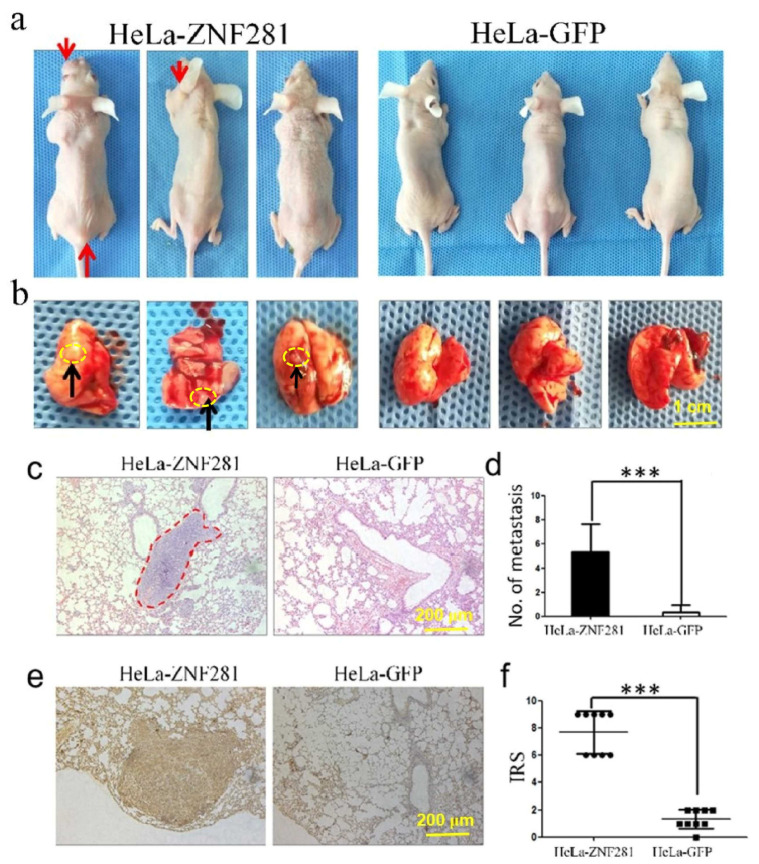
ZNF281 overexpression promoted affect HeLa cells lung metastasis in nude mice. (**a**) In nude mice injected with HeLa-ZNF281 and HeLa-GFP cells through the tail vein, the red arrow marks the subcutaneous mass of nude mice. (**b**) Lung tissues separated from the nude mouse (black arrow marked as lung surface bump). (**c**) Nude mouse lung tissue was stained with HE, and the red dotted line marked the tumor tissue. (**d**) The tumor lesion count in the lungs of nude mice injected with HeLa-ZNF281 cells and HeLa-GFP cells in groups was performed by *t*-test. (**e**) Lung tissues of nude mice with HeLa-ZNF281 cells and HeLa-GFP cells stained with ZNF281 by immunohistochemistry. (**f**) Immunoreactivity score (IRS) of lung tissues of nude mice. Statistical analysis was performed using two-tailed unpaired Student’s *t*-test. *** *p* < 0.001.

To investigate the effect of ZNF281 on metastasis, HeLa-GFP or HeLa-ZNF281 cells were injected into the tail vein. After 90 days, the mice were sacrificed and lung tissues were removed. Mice injected with HeLa-ZNF281 cells showed tumor growth, whereas those injected with control did not show any abnormalities (Figure 5a,b). H&E staining showed a significantly higher number of tumor lesions in the lung of mice injected with HeLa-ZNF281 cells compared with control. In addition, HE staining showed that the expression level of ZNF281 in the lung of mice injected with HeLa-ZNF281 cells was significantly higher than control (Figure 5c,d). Immunoreactivity scores (IRS) were also higher at metastasis sites of HeLa-ZNF281-injected mice than HeLa-GFP-injected mice (Figure 5e,f). These results indicate that ZNF281 promotes the metastasis of CC cells in vivo.

### 3.6. Effect of ZNF281 in Expression of EMT-Related Genes

**Figure 6:**
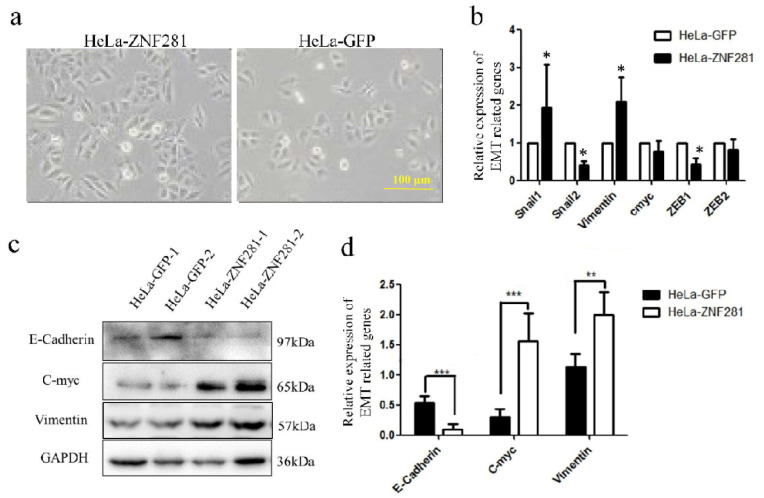
ZNF281 changes EMT-related genes. (**a**) Cell morphology comparisons of HeLa-ZNF281 and HeLa-GFP. (**b**) Real-time PCR was used to detect the transcription status of EMT-related genes of Snail1, Snail2, Vimentin, C-myc, ZEB1, and ZEB2. (**c**) Western blot analysis was performed to detect the expression of EMT-related genes in HeLa-ZNF281 cells and HeLa-GFP cells. (**d**) Band intensity of Western blot results. Statistical analysis was performed using two-tailed unpaired Student’s *t*-test. n = 6 dishes from 3 independent experiments. * *p* < 0.05, ** *p* < 0.01, and *** *p* < 0.001. Original western blots are presented in Figure S4.

EMT is crucial in the progression, invasion, and metastasis of CC, characterized by the loss of epithelial markers (e.g., E-cadherin) and the gain of mesenchymal markers (e.g., vimentin) [25]. In our study, HeLa-ZNF281 cells exhibited fusiform morphology (Figure 6a), suggesting EMT. Real-time PCR and Western blot analyses showed significantly increased *SNAI1* and *VIM* mRNA levels and significantly decreased *SNAI2* and *ZEB1* mRNA levels in HeLa-ZNF281 cells (Figure 6b). In addition, the level of E-Cadherin decreased, whereas the level of C-Myc and Vimentin increased in HeLa-ZNF281 cells compared with controls. These results indicate that ZNF281 promotes the de-epithelialization and EMT process in CC cells.

## 4. Discussion

CC is the second most common cancer in women in developing countries after breast cancer [26]. Prospective epidemiologic studies established the temporal association between exposure to high-risk HPV and the subsequent development of CC. These data indicated HPV as a necessary but insufficient cause of nearly 100% of CCs. The majority (~90%) of newly acquired HPV infections similarly become undetectable within 1–2 years [27], a phenomenon routinely described as “viral clearance” but which may also represent immune control below detectable levels or viral latency [28,29]. A detectable immune response is generated approximately 60% of the time [30], evidenced by the presence of serum antibodies specific to the HPV type causing infection, with uncertain ability to provide immunity against re-infection [31]. A minority of HPV infections are persistently detected beyond 12 months, increasing the risk of carcinogenic progression to cervical pre-cancer and potentially cancer if untreated [4]. For the molecular mechanisms, multistep factors such as proto-oncogene activation, tumor suppressor gene inactivation, epigenetic alterations, and genomic instability are involved in the development of CC [32,33]. Evidence indicates that various oncogenes (e.g., *LGR5* and *DAX1*) and suppressor genes (e.g., *KLF4* and *SLUG*) exhibit abnormal expression during cervical carcinoma progression [34], highlighting the importance of genetic factors.

The *ZNF281* gene is relatively conserved during mammalian evolution and is located on the long arm of chromosome 1 in humans [8]. As a transcription factor belonging to the Kruppel zinc finger family, it has four C2H2 zinc finger domains (amino acid residues 263–368) separated by the STGREKRPFY common sequence [8]. ZNF281 can both promote and suppress transcriptions of its target genes. ZNF281 directly activates the expression of gastrin and HIS3 but inhibits ornithine decarboxylase by binding to a GC-rich promoter sequence [35]. High expression of ZNF281 after surgical resection of a colorectal tumor correlates with a higher recurrence rate 3 years later [36]. Here, we also found a higher ZNF281 level in the CC group than in the NC group. Moreover, in some NC samples, ZNF281 was as high as CC samples because of unknown reasons, which needs further research. It was upregulated in most types of cancer and related to a worse prognosis in several tumors [17]. Increased expression of ZNF281 in tumors is partly due to the methylation of CpG or mutation of p53. It plays a role in the regulation of embryonic stem cell (ESC) differentiation and is very important in maintaining cellular stemness [37]. Knock-out of ZNF281 induces multipotent stem cell differentiation to osteogenic lineage [20]. Moreover, EMT is activated by ZNF281 in colon cancer cells through the regulation of *SNAI1* and other EMT-related gene expressions [38]. Besides the ZNF281 protein, long non-coding RNA (lncRNA) ZNF281 was also reported to be involved in the malignant progression of various malignant disorders, including cervical carcinoma, by targeting Kruppel-like factor 15 (*KLF15*) [39]. The relationship between the lncRNA ZNF281 and ZNF281 protein needs to be proven.

In this study, we found no effect of ZNF281 overexpression on the proliferation of CC cells. However, trans-well experiments showed that ZNF281 facilitates migration and invasion in vitro. On the one hand, according to previous findings, it is suggested that ZNF281 is closely related to EMT [40,41]. Multiple signal pathways related to EMT interact to form a complex network. RT-PCR showed significantly decreased *SNAI2* (Slug) mRNA level and increased *SNAI1* mRNA level in ZNF281-overexpressing cells. Previous studies showed that Snail1 and ZNF281 promote the expression of each other and jointly promote the EMT process [41]. As Snail1 and Snail2 proteins are closely related, their functions overlap, leading to competitive interaction [42,43]. We found increased C-myc and vimentin, two markers participating in the EMT process [44,45], in HeLa-ZNF281 cells. However, the effect of ZNF281 on *MYC* has not been explained and requires further experimental observation. And the inconsistency results of *MYC* mRNA and protein after ZNF281 overexpression indicate some post-translational mechanisms were also regulated by ZNF281. E-cadherin was significantly decreased in the HeLa-ZNF281 cells and had lost its original epithelial phenotype. These findings confirm the role of ZNF281 in facilitating EMT.

## 5. Conclusions

We found that ZNF281 promoted metastasis in HeLa cell lines by promoting the EMT process, thus facilitating the transfer of HeLa cell lines in vivo. Our research might provide a new marker for predicting the development of CC.

## Data Availability

The datasets used and/or analyzed during the current study are available from the corresponding authors on reasonable request.

## Supplementary Materials

The following supporting information can be downloaded at: https://www.mdpi.com/article/10.3390/cancers16213717/s1, Figure S1: Full size blots of Figure 1a; Figure S2: Full size blots of Figure 1c; Figure S3: Full size blots of Figure 2d; Figure S4: Full size blots of Figure 6c.
