# Supplementary material for: ZNF281 Facilitates the Invasion of Cervical Cancer Cell Both In Vivo and In Vitro
Source: Cancers (Basel). 2024 Nov 4;16(21):3717. doi: 10.3390/cancers16213717 (PMC11545007; doi:10.3390/cancers16213717)
Supplement: Supplementary file 1 [file cancers-16-03717-s001.zip › cancers-3268854-supplementary.pdf]

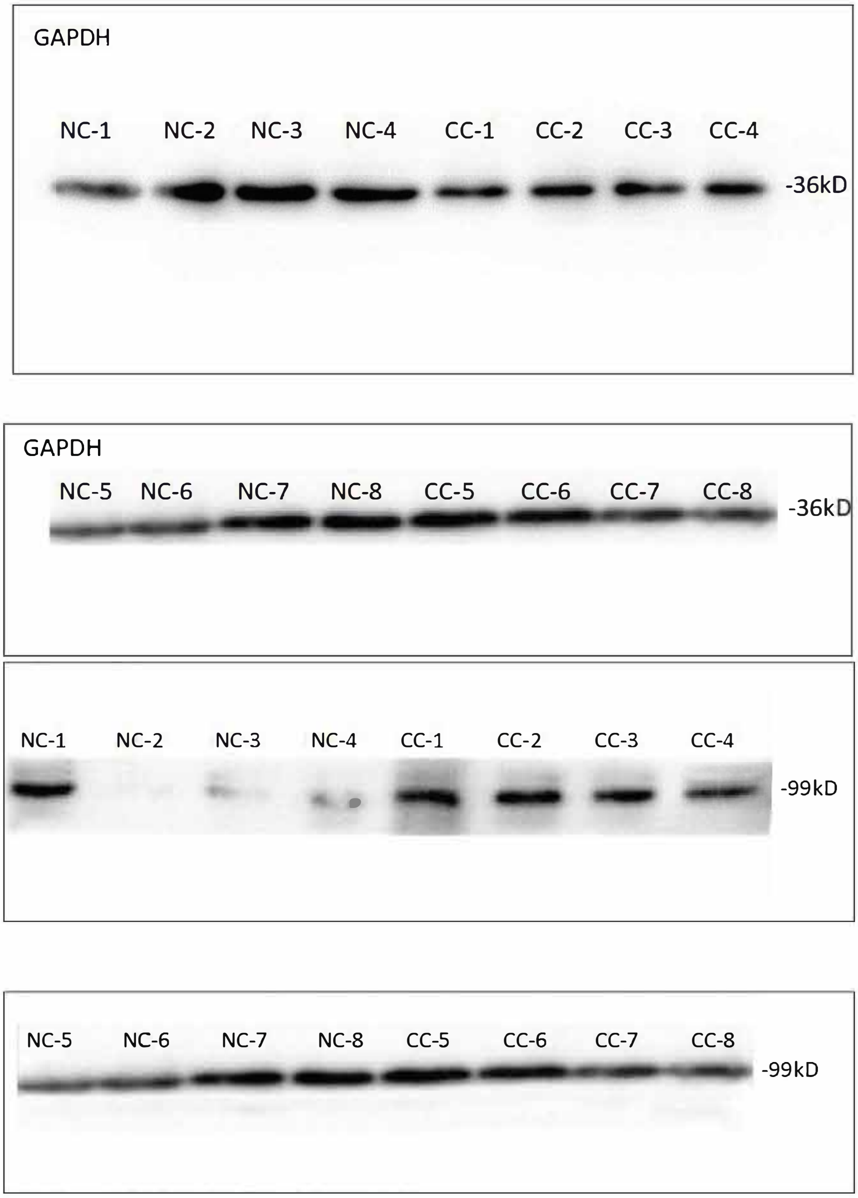

Figure S1: Full size blots of Figure 1a.

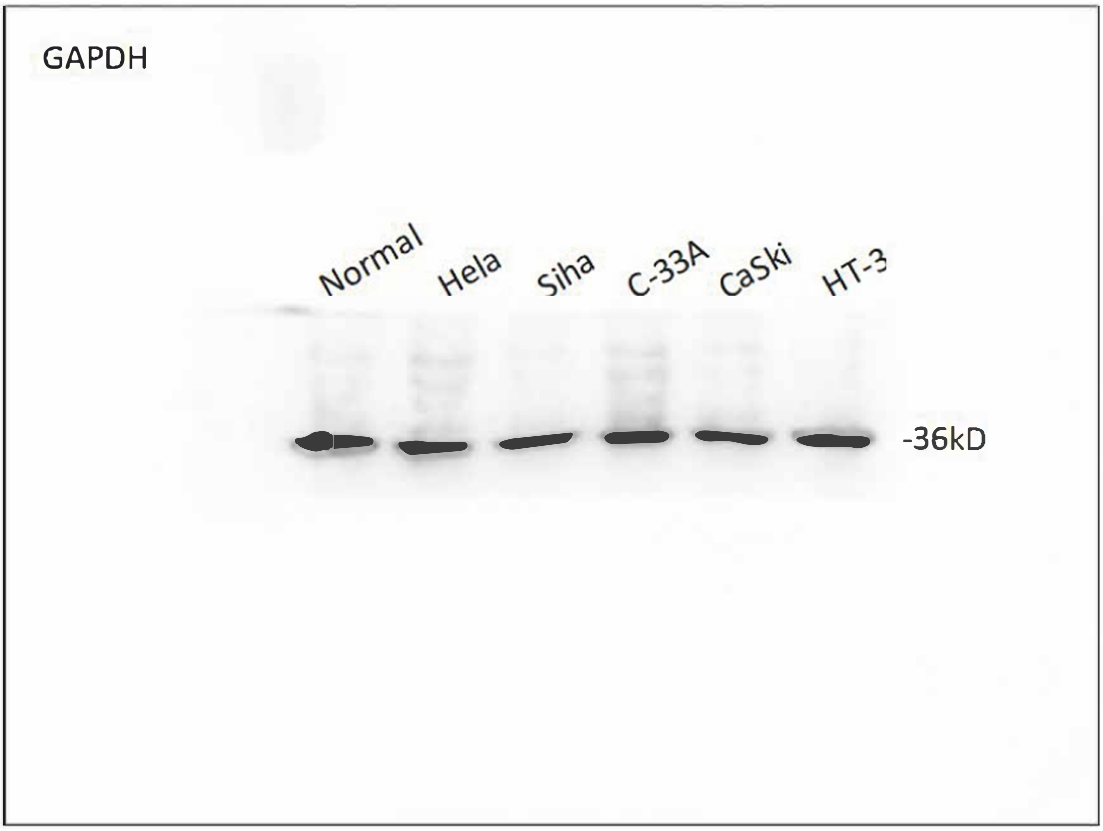

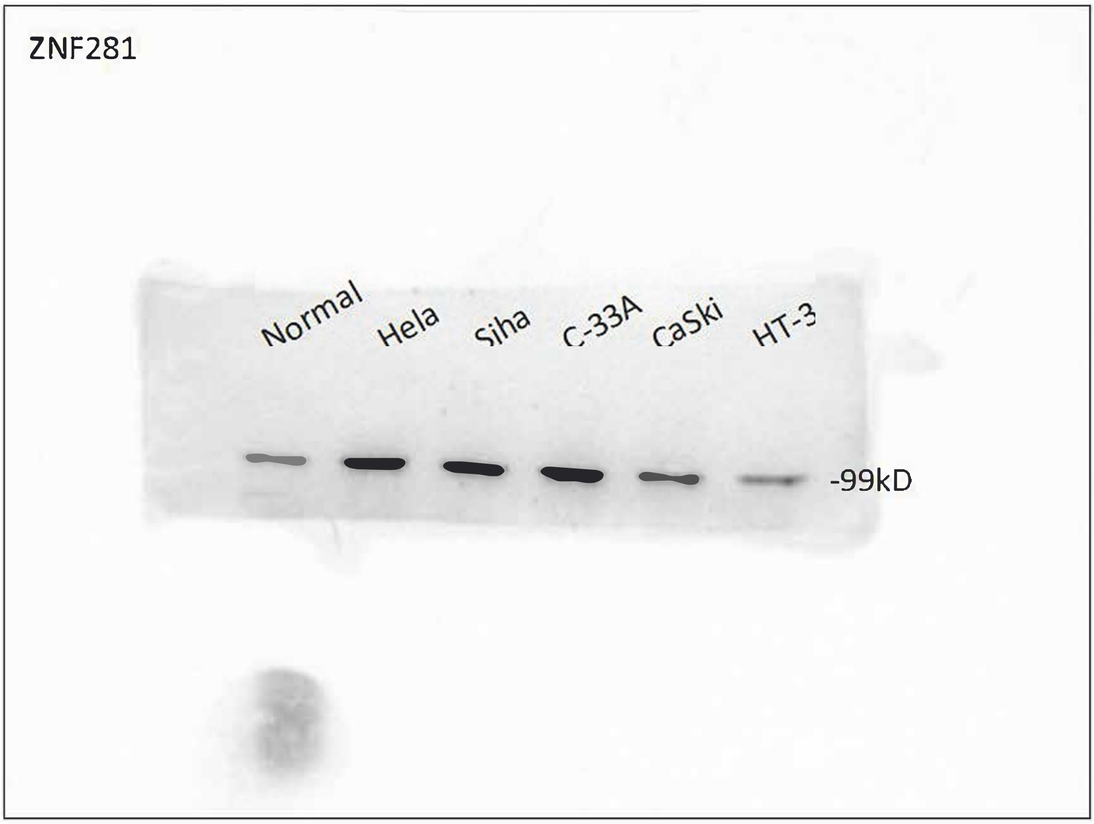

Figure S2: Full size blots of Figure 1c.

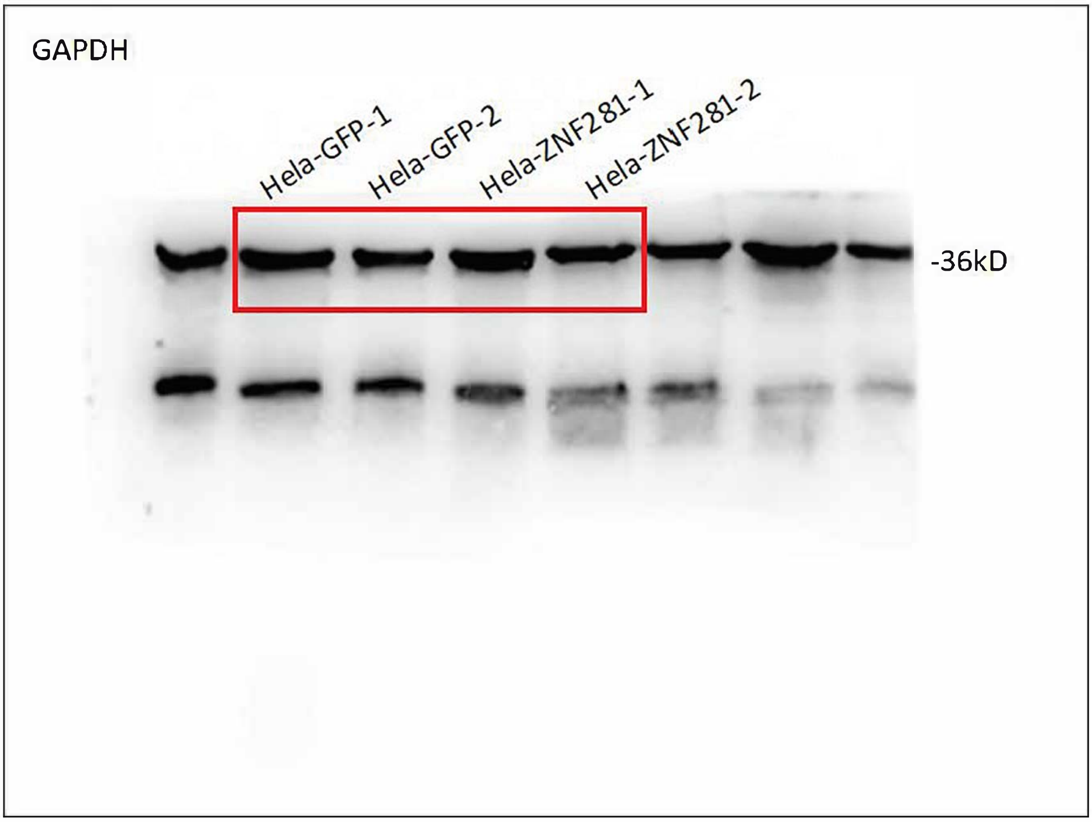

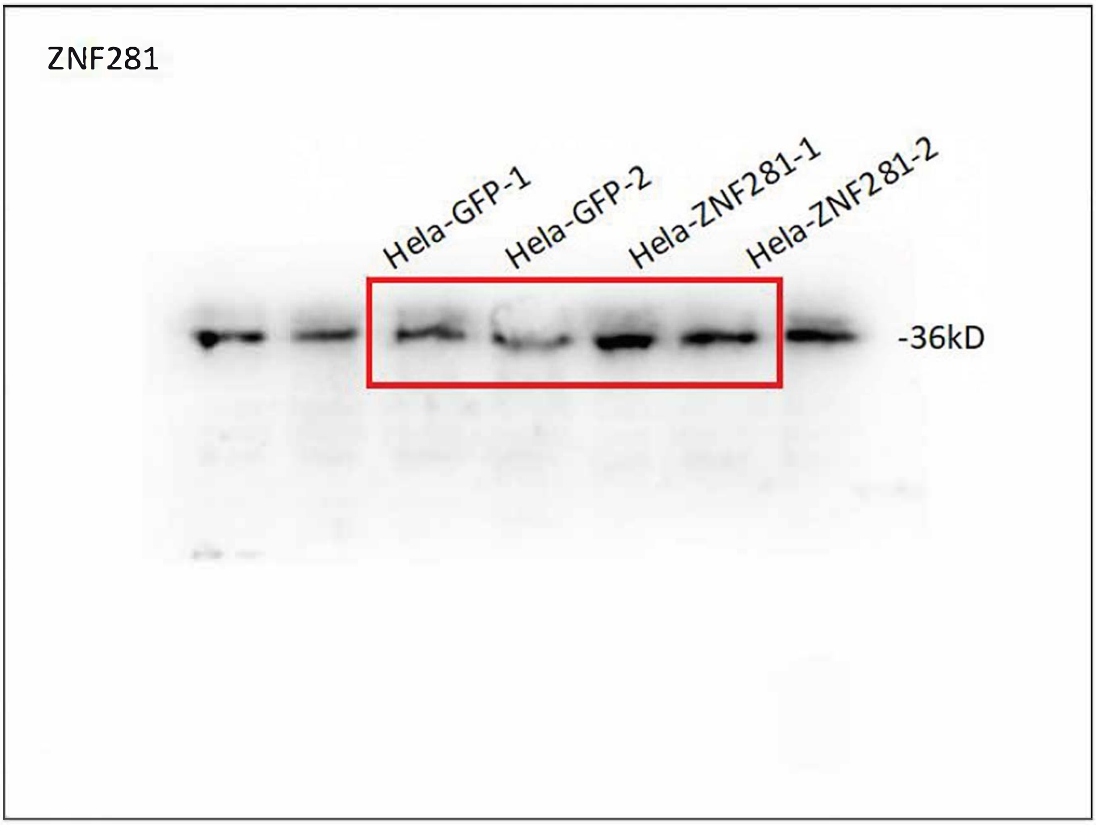

Figure S3: Full size blots of Figure 2d.

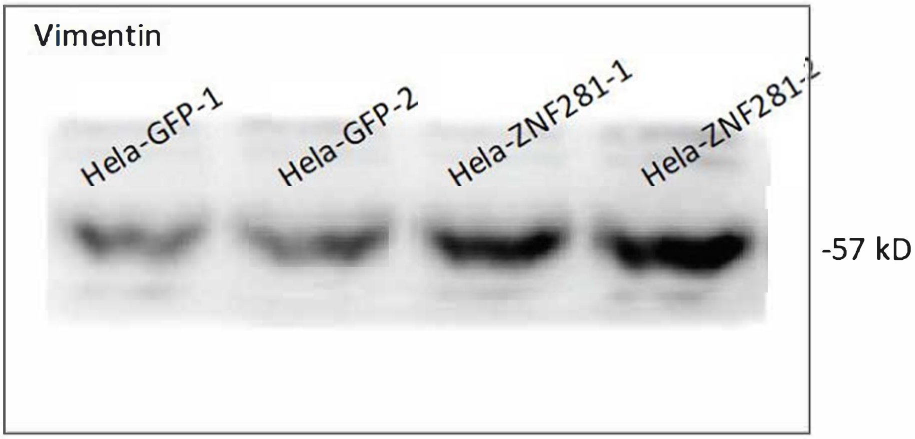

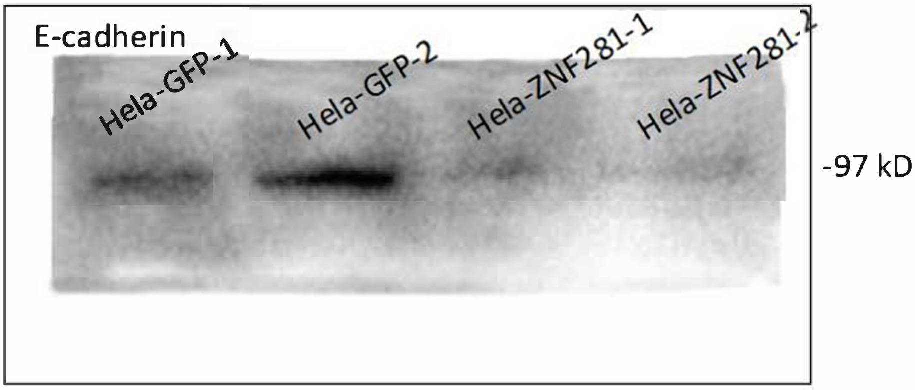

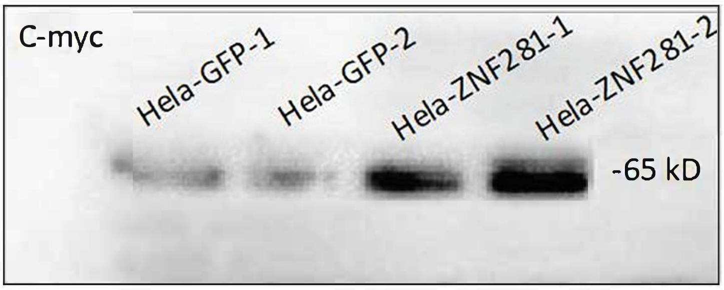

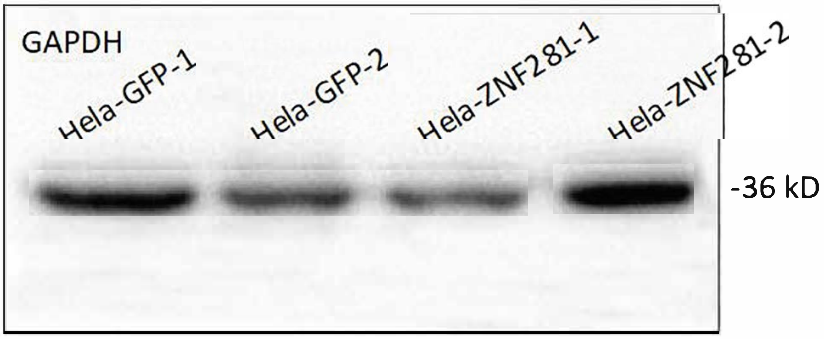

Figure S4: Full size blots of Figure 6c.
